# Identification of potential diagnostic gene biomarkers in patients with osteoarthritis

**DOI:** 10.1038/s41598-020-70596-9

**Published:** 2020-08-12

**Authors:** Xinling Wang, Yang Yu, Yong Huang, Mingshuang Zhu, Rigao Chen, Zhanghui Liao, Shipeng Yang

**Affiliations:** Hospital of Chengdu University of Traditional Chinese Medicine, No. 39, Shierqiao road, Jinniu District, Chengdu City, 610075 Sichuan Province China

**Keywords:** Molecular biology, Biomarkers, Rheumatology

## Abstract

The current study was aimed to identify diagnostic gene signature for osteoarthritis (OA). The differentially expressed genes (DEGs) in synovial membrane samples and blood samples were respectively identified from the GEO dataset. The intersection DEGs between synovial membrane and blood were further screened out, followed by the functional annotation of these common DEGs. The optimal intersection gene biomarkers for OA diagnostics were determined. The GSE51588 dataset of articular cartilage was used for expression validation and further diagnostic analysis validation of identified gene biomarkers for OA diagnostics. There were 379 intersection DEGs were obtained between the synovial membrane and blood samples of OA. 22 DEGs had a diagnostic value for OA. After further screening, a total of 9 DEGs including TLR7, RTP4, CRIP1, ZNF688, TOP1, EIF1AY, RAB2A, ZNF281 and UIMC1 were identified for OA diagnostic. The identified DEGs could be considered as potential diagnostic biomarkers for OA.

## Introduction

Osteoarthritis (OA) is a chronic joint disease featured with cartilage degeneration, synovial inflammation, osteophyte formation, and subchondral bone sclerosis^1^. Typical symptoms of OA include pain, swelling, and stiffness, and often accompanied by dysfunction and limited mobility^2^. Abnormal mechanical stress, aging, obesity and genetic factors are common risk factors for OA progression^3^. In the previous study, most researchers focused on the role of cartilage tissue and chondrocytes in OA mechanisms and treatment^4^. In recent years, with the further development of medicine, synovial tissue and blood have been explored. Previous studies reported that synovial tissue played a crucial role in OA^5,6^. The synovial membrane produces and regulates synovial fluid, maintains joint activity, and is adversely affected in joint diseases as part of the joint structure^7^. Synovial lesions can also be found in many joint diseases, which may play an important role in promoting the development and progression of the disease. In addition, synovial membrane and peripheral blood have been used for pathological analysis in end-stage osteoarthritis knee joints^8^.

In recent years, more and more researchers have performed the study of the molecular characteristics of OA. Among which, high-throughput microarray methods have received extensive attention and have made great progress in the fields of molecular diagnosis and classification, prognosis prediction, and discovery of target drug^9,10^. In previous studies, several gene expression profiling studies of OA have revealed several key genes, diagnostic gene biomarkers and enriched signaling pathways of these genes^11,12^. In addition, biomarkers could help identify early degradation in OA, and may be applied to decision making in the clinical practice^13,14^. If potential diagnostic molecular markers of OA before the disease could be screened out^15^, the life quality of patients could be improved.

In order to explore the molecular mechanism and identify potential diagnostic gene biomarkers for OA, the high-throughput transcriptome data of OA from the synovial membrane and blood samples were firstly analyzed based on the GEO datasets. The intersection DEGs between synovial membrane and blood samples were further identified. Then, machine learning was used to for identification of diagnostics intersection DEGs for OA. Finally, the selected DEGs were verified in expression level and diagnostic capability using OA data from articular cartilage in the GEO database.

## Materials and methods

### Microarray data information

The Gene Expression Omnibus (GEO, https://www.ncbi.nlm.nih.gov/geo) is a public genomics data repository that stores gene expression profiles, raw series and platform records. 4 datasets of synovial membrane including GSE82107, GSE55457, GSE55235 and GSE12021 and 2 datasets of blood including GSE63359 and GSE48556 were used for gene expression profiles analysis. All these datasets were downloaded from the GEO database, which based on GPL 570, GPL96 and GPL6947 platforms. Details of above six datasets were listed in Table 1. In addition, the clinical information of patients in these datasets was shown in Table 2.

**Table 1:** Six datasets used for gene expression profiles analysis.

GEO ID	Author	Platform	Samples	Year	Type	Omics
GSE82107	Marieke de Vries	GPL 570	OA: Normal = 10:7	2016	Synovial membrane	mRNA
GSE55457	Woetzel D	GPL 96	OA: Normal = 10:10	2014	Synovial membrane	mRNA
GSE55235	Woetzel D	GPL 96	OA: Normal = 10:10	2014	Synovial membrane	mRNA
GSE12021	Huber R	GPL 96	OA: Normal = 10:9	2008	Synovial membrane	mRNA
GSE63359	Attur M	GPL 96	OA: Normal = 44:26	2017	Blood	mRNA
GSE48556	Ramos YF	GPL 6,947	OA: Normal = 106:33	2013	Blood	mRNA

*OA* osteoarthritis.

**Table 2:** The clinical information of patients in datasets.

	OA	Normal
**GSE82107**	10	7
Age, years (mean ± SD)	NA	NA
Sex		
Male	NA	NA
Female	NA	NA
**GSE55457**	10	10
Age, years (mean ± SD)	72.4 ± 5.9	51.0 ± 19.7
Sex		
Male	2	8
Female	8	2
**GSE55235**	10	10
Sex		
Male	2	7
Female	8	3
**GSE12021**	10	9
Age, years (mean ± SD)	71.9 ± 2.0	49.9 ± 6.7
Sex		
Male	2	7
Female	8	2
Disease duration, years (mean ± SD)	6.2 ± 2.7	0.4 ± 0.3
CRP(mg/L)	7.6 ± 2.9	NA
**GSE63359**	46	26
Age, years (mean ± SD)	65.6 ± 10.7	54.6 ± 9.5
Sex		
Male	14	7
Female	32	19
BMI (mean ± SD)	27.4 ± 4.1	24.9 ± 3.9
**GSE48556**	106	33
Age, years (mean ± SD)	58.2 ± 7.6	60.9 ± 7.7
Sex		
Male	1	9
Female	105	24
**GSE51588**	40	10
Age, years (mean ± SD)	69.7 ± 8.8	38.4 ± 12.7
Sex		
Male	18	4
Female	22	6

### Data processing and identification of DEGs

The process of data preprocessing included background adjustment, normalization, and summarization. The raw data was preprocessed by affy package in R software. The limma package in R software was used to identify the DEGs in OA synovial membrane and blood samples. Benjamini and Hochberg test was used to adjust the p value. The *p* < 0.05 was considered as the cutoff criterion. The intersection DEGs between synovial membrane and blood samples was shown by the Venn diagram (https://bioinfogp.cnb.csic.es/tools/venny/index.html).

### Functional annotation analysis of intersection DEGs

GOplot package in R software was used for Gene Ontology (GO) and Kyoto Encyclopedia of Genes and Genomes (KEGG, www.kegg.jp/kegg/kegg1.html) enrichment analysis of intersection of DEGs was performed through GeneCoDis3 (https://genecodis.cnb.csic.es/analysis) software. *p* < 0.05 was considered to indicate a statistically significant GO and KEGG terms.

### Identification of the optimal diagnostic gene biomarkers for OA

In order to identify optimal diagnostic gene biomarkers for OA, we utilized the interaction DEGs obtained between synovial membrane and blood samples as feature variables to establish the model. However, all these DEGs have serious redundant information. The LASSO package in R software^16^ algorithm analysis was performed by using the ‘glmnet’ package (https://cran.r-project.org/web/packages/glmnet/) to reduce data dimensions. To further identify the optimal diagnostic gene biomarkers for OA, feature selection procedures were performed as previously described^17^. The random forest package in R was used to build the random forest model. The ‘rpart’ package in R (https://cran.r-project.org/web/packages/rpart/) was used to build the decision tree model. The e1071 package (https://cran.r-project.org/web/packages/e1071/index.html) in R was used to establish the support vector machine (SVM) model. The diagnostic ability of above three models and each gene biomarker was evaluated by the receiver operating characteristic (ROC) area under the curve (AUC), sensitivity and specificity.

### In silico* validation of DEGs using GSE51588*

The GEO database (GSE51588) was used to validate the expression of selected intersection DEGs. The sample in the GSE51588 dataset was articular cartilage tissues from 40 patients with OA and 10 normal controls. The ggpubr, magrittr and ggsignif package in R software was used for analysis. The expression level of selected intersection DEGs was presented as box-plots. In addition, the GSE51588 dataset was used to further validate the diagnostic capacity of intersection DEGs identified in the diagnostic model analysis. In this process, pROC package in R software was used for analysis.

### QRT-PCR validation and statistical analysis

In this study, 4 patients with OA and 4 normal controls were enrolled in the study. The blood, joint fluid and cartilage tissue samples of these individuals were further collected. The inclusion criteria of OA patient was as follows: (1) patients were diagnosed early OA according to the diagnostic criteria; (2) blood was collected before immunoregulatory therapy with glucocorticoid. The exclusion criteria of OA patient was as follows: (1) patients with other inflammatory arthritis or autoimmune diseases, including rheumatoid arthritis, gout, systemic lupus erythematosus and so on; (2) patients with a history of severe knee trauma (Kellgren-Lawrance classification: IV); (3) patients had a history of steroid injection or use of non-steroid drugs within the past 3 months; (4) patients with severe liver and kidney diseases (three times higher than normal value) and cardiovascular diseases (classification of cardiac function in New York heart association: IV). QRT-PCR was used to validate the expression level of TLR7, CRIP1, TOP1 and UIMC1. The details of qRT-PCR were performed as previously described^1^. The written informed consent was obtained from all individuals. In addition, the study was approved by the ethics committee of the hospital of Chengdu University of Traditional Chinese Medicine. All research was performed in accordance with relevant guidelines/regulations.

## Results

### Identification of DEGs in OA

**Figure 1:**
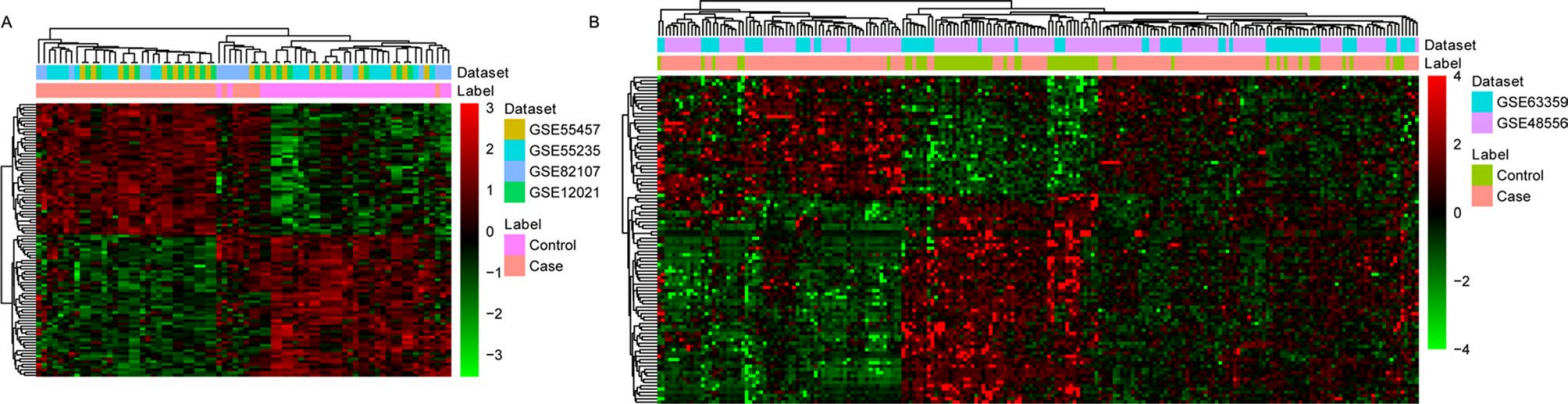
Unsupervised hierarchical clustering analysis showing expression profiles of top 100 DEGs in synovial membranes of samples (**A**), and blood samples (**B**). Row and column represented DEGs and samples, respectively. The color scale indicated the expression of DEGs.

**Figure 2:**
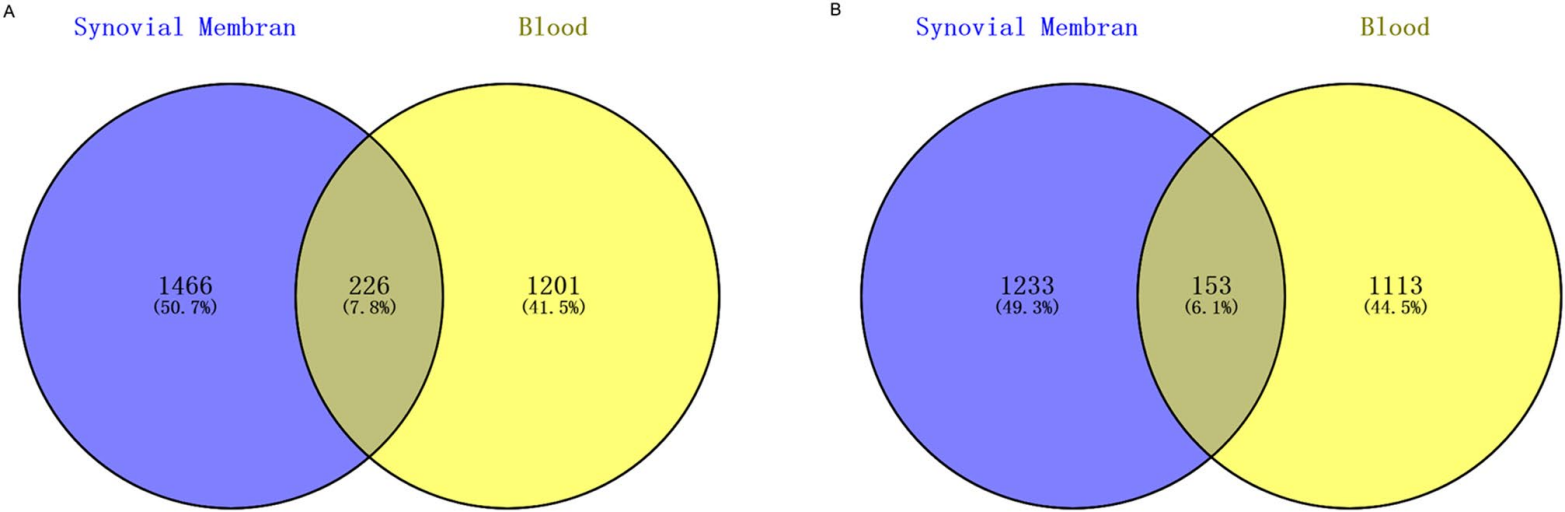
Venn diagrams showing the overlap of DEGs between synovial membrane samples and blood samples. Numbers represented the number of DEGs. Percentage was represented by the ratio of current DEGs to total. (**A**) up-regulated DEGs, (**B**) down-regulated DEGs. Venn diagram was drew by the online tool (https://bioinfogp.cnb.csic.es/tools/venny/index.html).

By integrated analysis, a total of 3,078 (1692 up-regulated and 1,386 down-regulated) DEGs were identified in the synovial membrane sample of OA. Similarly, a total of 3,078 (1,427 up-regulated and 1,266 down-regulated) DEGs were identified in the blood sample of OA. Hierarchical clustering analysis of the top 100 DEGs in synovial membrane and top 100 DEGs in blood was presented in Fig. 1A,B, respectively. In addition, a total of 379 intersection DEGs were obtained between the synovial membrane and blood samples (Fig. 2).

### Functional annotation of intersection DEGs

**Figure 3:**
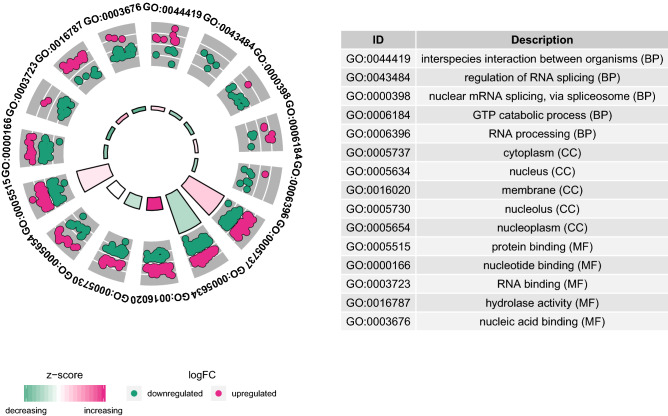
Top 5 significant enrichment GO terms of intersection DEGs. The z-score clustering in the GO terms of intersection of DEGs was shown below. The red and green color represents up-regulated and down-regulated DEG, respectively. *BP* biological process, *CC* cellular component, *MF* molecular function. GOplot package in R software was used for GO analysis.

**Figure 4:**
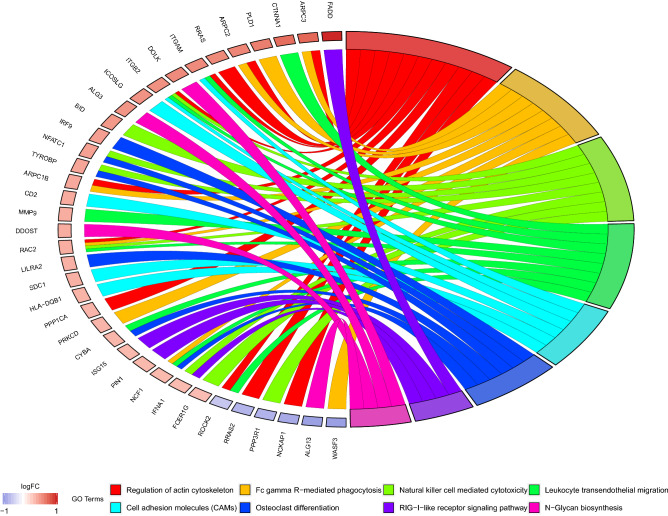
Top 8 KEGG signaling pathways of intersection DEGs. GOplot package in R software was used for KEGG analysis.

GO enrichment analysis and the KEGG pathway analysis manifested that these common DEGs were significantly involved in the GO items of regulation of interspecies interaction between organisms, cytoplasm and protein binding (Fig. 3). In addition, regulation of actin cytoskeleton was the most significant enriched KEGG pathway (Fig. 4).

### Identification of the optimal diagnostic gene biomarkers for OA

**Figure 5:**
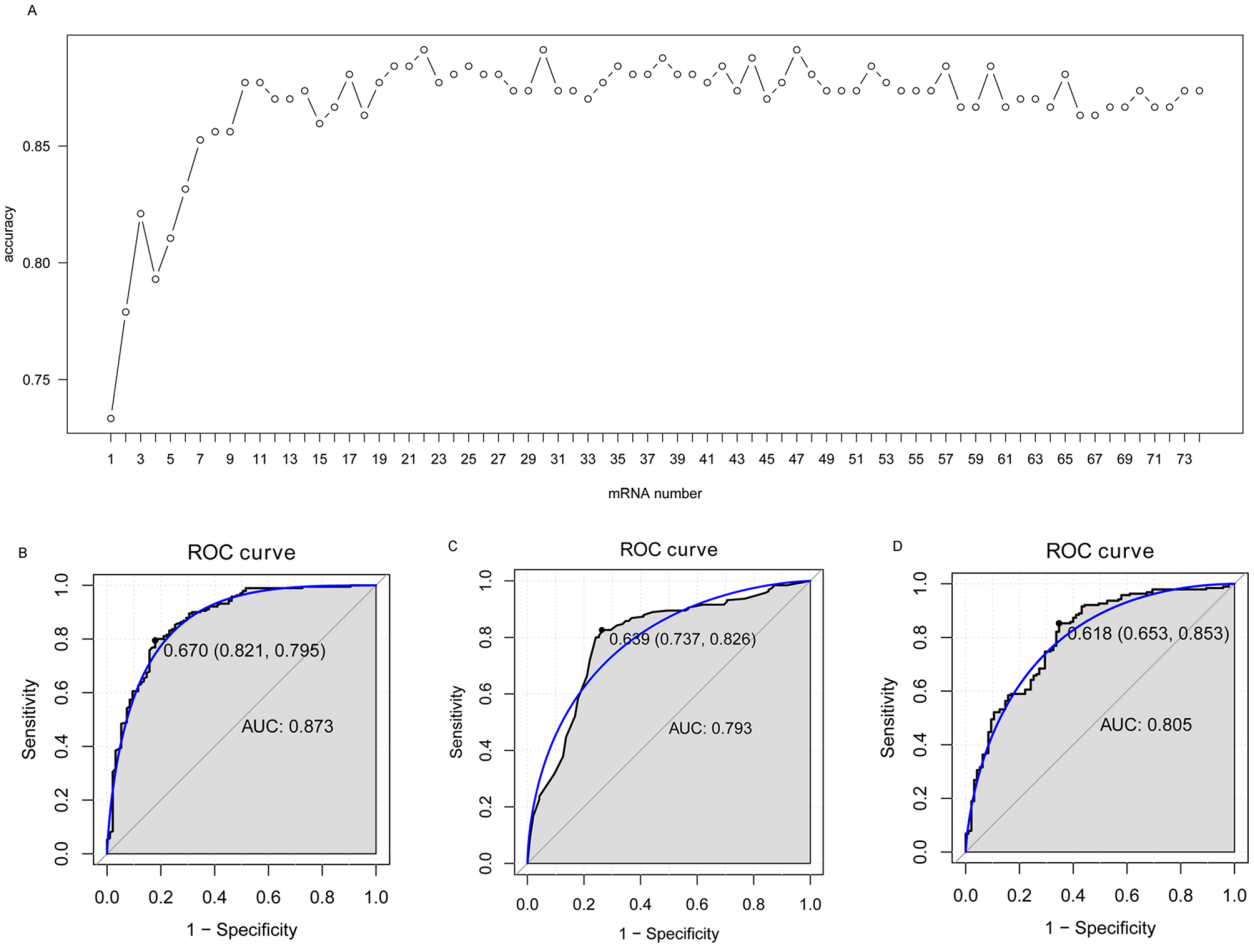
Identification of gene biomarkers from intersection DEGs for OA. (**A**) Variance rate of classification performance when increasing numbers of the predictive DEGs. (**B**) ROC analysis of 22 OA-specific gene biomarkers. The LASSO package in R software, the random forest package in R software, the ‘rpart’ package in R software, the e1071 package in R software were used for analysis.

We identified 74 DEGs using LASSO algorithm analysis from 379 intersection DEGs. These 74 DEGs were ranked by using the random forest analysis, according to the decrease in mean accuracy (Fig. 5). The average accuracy rate of top 22 DEGs showed the highest score due to the tenfold cross-validation result. Therefore, we selected these 22 DEGs as the optimal potential diagnostic gene biomarkers for OA. The AUC of the random forests model, decision tree model and SVM model was 0.873, 0.793 and 0.805, respectively (Fig. 5B).

### Validation of diagnostic gene biomarkers in GSE51588 dataset

**Figure 6:**
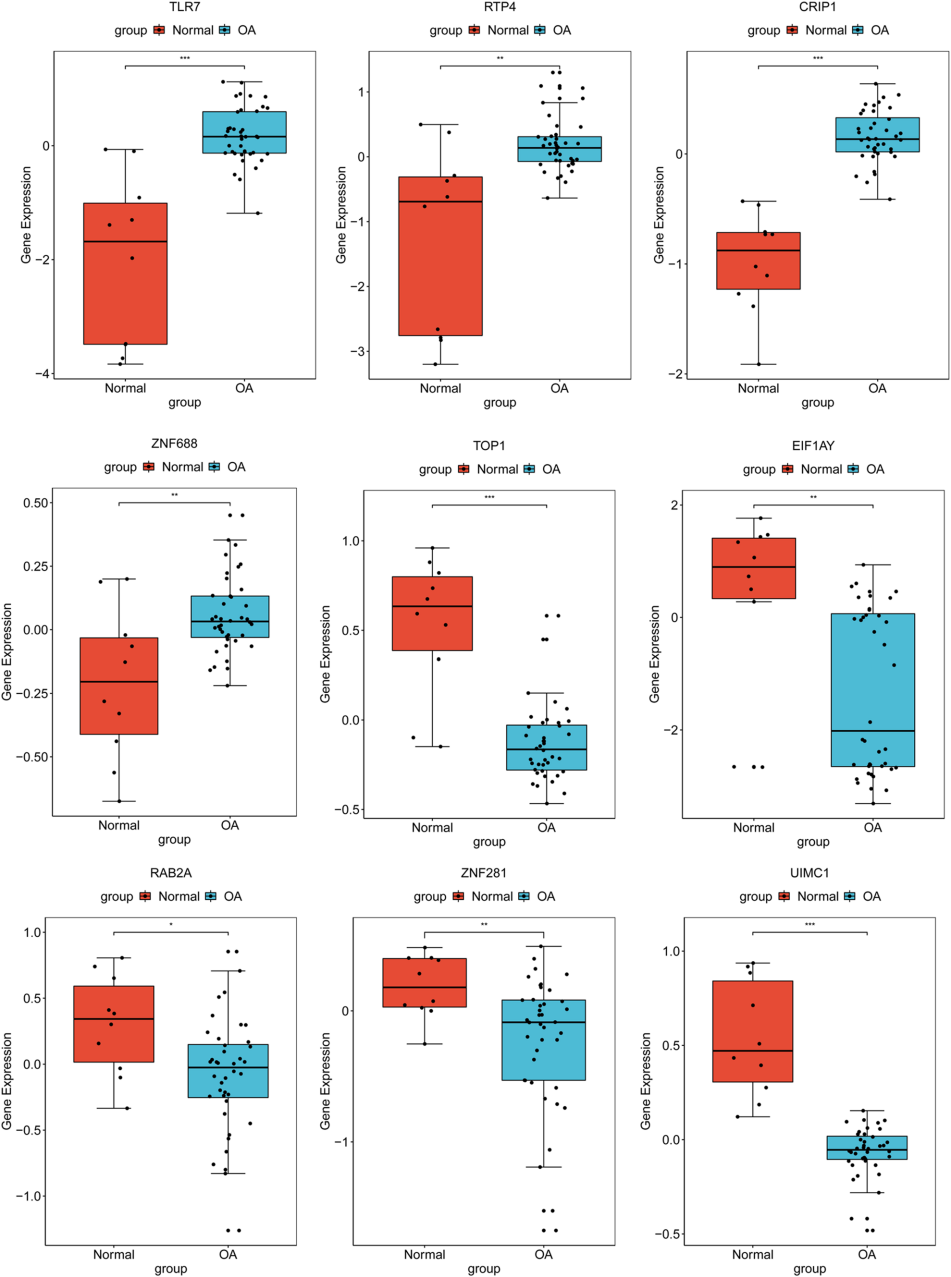
Validation of the expression levels of identified 9 diagnostic gene biomarkers for OA in GSE51588 dataset. The x-axis showed OA and normal groups, and y-axis showed gene expression level. *p < 0.05; **p < 0.01; ***p < 0.001. The ggpubr, magrittr and ggsignif package in R software was used for analysis.

**Figure 7:**
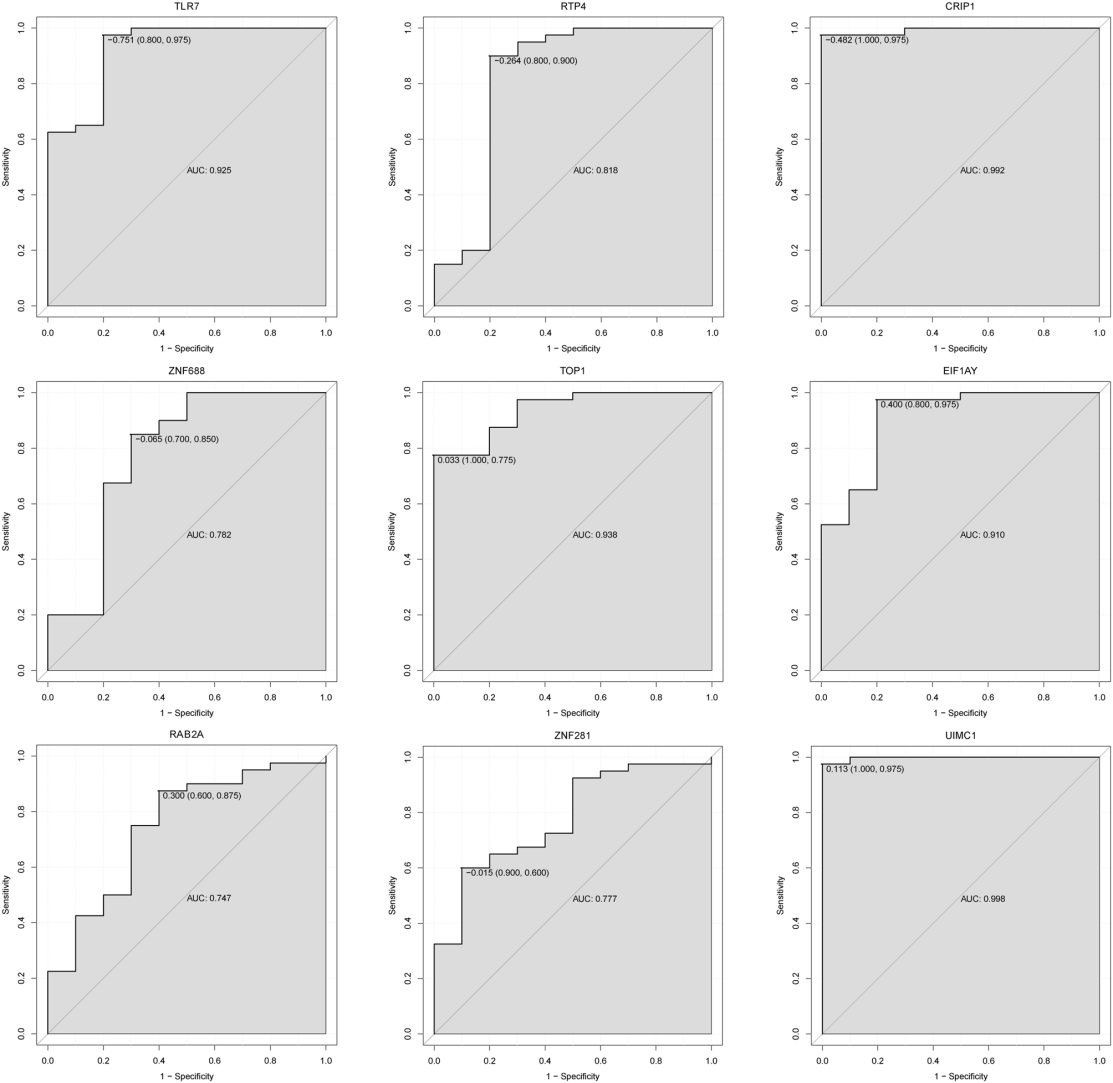
The ROC curves of identified 9 diagnostic gene biomarkers in the GSE51588 dataset. The ROC curves were used to show the diagnostic ability with 1-specificity and sensitivity. The pROC package in R software was used for analysis.

After further screening (consistent expression pattern) in GSE51588 dataset from 22 diagnostic gene biomarkers for OA, a total of 9 DEGs including TLR7, RTP4, CRIP1, ZNF688, TOP1, EIF1AY, RAB2A, ZNF281 and UIMC1 were finally identified. The expression pattern of these DEGs in the GSE51588 dataset was shown in Fig. 6. TOP1, EIF1AY, RAB2A, ZNF281 and UIMC1 were significantly down-regulated. TLR7, RTP4, CRIP1 and ZNF688 were remarkably up-regulated. The result was consistent with the integrated analysis in the synovial membrane and blood samples. In addition, we also performed the diagnostic capability validation of above 9 DEGs in the GSE51588 dataset. Significantly, AUC values of these 9 DEGs were all > 0.7, which suggested that they have potential diagnostic value for OA (Fig. 7).

### QRT-PCR validation of diagnostic gene biomarkers

**Figure 8:**
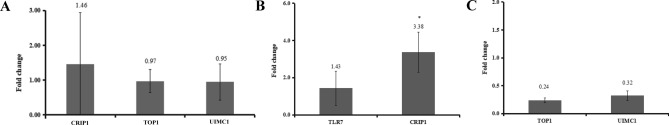
QRT-PCR verification results of CRIP1, TOP1, TLR7 and UIMC1 in blood (**A**), joint fluid (**B**) and cartilage tissue (**C**) samples of OA. The x-axis was indicated different samples, and the y-axis was indicated the relative expression level of the gene. *p < 0.05.

In this study, 4 patients with OA and 4 normal individuals were enrolled for blood (CRIP1, TOP1 and UIMC1), joint fluid (TLR7 and CRIP1) and cartilage tissue (TOP1 and UIMC1) analysis. The clinical information of OA patients was listed in Table 3. In blood, the expression of TOP1 and UIMC1 was down-regulated, while the expression of CRIP1 was up-regulated (Fig. 8A). In joint fluid, the expression of TLR7 and CRIP1 was up-regulated (Fig. 8B). In cartilage tissue, the expression of TOP1 and UIMC1 was down-regulated (Fig. 8C). The result was consistent with results of our integrated analysis.

**Table 3:** The clinical information of OA patients normal individuals in QRT-PCR.

	Age	Gender	BMI	Duration of disease	Kellgren-Lawrance classification	Site	White blood cell count (cells/ml)	Platelet count (cells/ml)	C-reactive protein (mg/l)	X-ray results
OA	55	Female	25.4	30 years	I	Left knee	5.83 × 10^9^	2.55 × 10^11^	< 0.5	Mild degeneration of the left knee joint
	29	Male	24.8	2 months	I	Left knee	4.58 × 10^9^	3.07 × 10^11^	< 0.5	Mild degeneration of the left knee joint
	65	Female	20	1 month	I	Left knee	2.01 × 10^9^	1.35 × 10^11^	< 0.5	Mild degeneration of the left knee joint
	76	Male	28.3	5 years	III	Right knee	8.36 × 10^9^	2.16 × 10^11^	< 0.5	Degenerative changes in both knees
NA	60	Female	24	3 days	I	Left knee	5.33 × 10^9^	9.5 × 10^11^	9.5	Patellar fractures
	48	Male	22	5 days	Normal	Right knee	7.8 × 10^9^	1.54 × 10^11^	11.1	Fracture of the upper tibia
	45	Male	27	1 days	Normal	Right knee	8.8 × 10^9^	1.87 × 10^11^	14.2	Platform fractures
	37	Female	24	1 days	Normal	Left knee	9.1 × 10^9^	2.01 × 10^11^	11.9	Patellar fractures

*OA* osteoarthritis, *NC* normal controls.

## Discussion

OA is the most common degenerative joint disease observed in the world, which puts a heavy burden on people’s health and medical insurance. Therefore, early diagnosis and treatment of OA are particularly important^1^. The role of cysteine-rich intestinal protein (CRIP) as a novel biomarker in several cancers has been widely reported^18–20^. But the function of CRIP1 in OA was rarely studied. CRIP belongs to the same group of proteins as cysteine-rich proteins, and is a member of the LIM/double zinc-finger protein^21^. It has a unique double zinc-finger motif as its defining feature^19^. Wang et al. found that CRIP1 was one of the differently expressed genes in non-traumatic osteonecrosis of femoral head cartilage^22^. The present study indicated that CRIP1 was up-regulated in the synovial membrane, blood and articular cartilage samples, which may be served as a diagnostic biomarker for OA.

The Toll-like receptor 7 (TLR7) plays an important role in pathogen recognition and activation of innate immunity. Previous studies have shown that TLR7 is expressed in rheumatoid arthritis monocyte derived dendritic cells and fibroblasts^23,24^. Elevated levels of TLR7 were found in rheumatoid arthritis lining and sublining macrophages^25^. It has been demonstrated that expression of TLR7 may be a predictor for rheumatoid arthritis disease activity, and targeting TLR7 may suppress chronic progression of rheumatoid arthritis^25^. It is noted that TLR7 is up-regulated in synovial membrane of RA patients compared with OA patients^24^. Our integrated analysis showed that in TLR7 was up-regulated in the synovial membrane, blood and articular cartilage samples of OA patients. Furthermore, in the functional enrichment analysis, we found that TLR7 was involved in the biological process of innate immune response, which was related to the development of OA. Previous studies indicated that the immune response is involved in the occurrence and development of OA^26,27^. In addition, we also found that TLR7 had a potential diagnostic value for OA. All these results suggested that TLR7 may play a crucial role in the pathogenesis of OA.

DNA topoisomerase I (TOP1) plays an important regulating action during osteoblast proliferation and migration^28,29^. The expression of eukaryotic translation initiation factor 1A Y-linked (EIF1AY) is decreased in bone marrow-derived very small embryonic-likecell^30^. In addition, EIF1AY is a differential gene in juvenile idiopathic arthritis^31^. RAB2A, member RAS oncogene family (RAB2A), located on articular chondrocyte, is associated with rheumatoid arthritis^32,33^. Zinc finger protein 281 (ZNF281), involved in bone metabolism, is related to osteoporosis and osteosarcoma^34–36^. Ubiquitin interaction motif containing 1 (UIMC1) is down-regulated in metastatic osteosarcoma^34^. Receptor transporter protein 4 (RTP4) is differentially expressed in rheumatoid arthritis^37^. Zinc finger protein 688 (ZNF688) is a lineage-specific transcription factor^38^. In the present study, we found that TOP1, EIF1AY, RAB2A, ZNF281 and UIMC1 were down-regulated, RTP4 and ZNF688 were up-regulated in the synovial membrane, blood and articular cartilage samples of OA patients. Furthermore, these genes all have a potential diagnostic value for OA.

Based on the KEGG pathway analysis, we found that was the most significantly enriched signaling pathway by intersection of DEGs. The role of the actin skeleton in the regulation of chondrocyte phenotype has been extensively studied. The differentiated chondrocytes have a cortical distribution of actin filaments. In the dedifferentiation of monolayer culture, primary chondrocytes lose the expression of cartilage matrix molecules (type II collagen and aggrecan) and express fibroblast matrix molecules, where obtaining elongation phenotype and forming actin stress fibers^39,40^.

In conclusion, the study indicated that the gene expression profiles were altered in synovial membrane, blood and articular cartilage samples of patients with OA. A total of 9 DEGs including TLR7, RTP4, CRIP1, ZNF688, TOP1, EIF1AY, RAB2A, ZNF281 and UIMC1 were identified for OA diagnostic. However, there are limitations to our study. Firstly, the sample in the QRT-PCR was small. Larger numbers of synovial membrane or blood or articular cartilage samples are further needed. Secondly, the potential molecular mechanism in cell or animal model is needed to explore the biological function of identified diagnostic gene biomarkers for OA.
